# Managementstrategie für den Massenanfall von Erkrankten/Infizierten in Alten- und Pflegeheimen im Kontext der COVID-19-Pandemie

**DOI:** 10.1007/s00063-021-00816-1

**Published:** 2021-04-20

**Authors:** Wolfgang Schreiber, Philipp Wolf, Nicole Bigalke, Marc U. Bigalke, Bernhard M. Graf, Michael S. Dittmar

**Affiliations:** 1 Zweckverband für Rettungsdienst und Feuerwehralarmierung Amberg, Amberg, Deutschland; 2 Klinik für Anästhesiologie, Universitätsklinikum Regensburg, Franz-Josef-Strauss-Allee 11, 93053 Regensburg, Deutschland; 3 Zentrale Notaufnahme, Klinikum St. Marien Amberg, Amberg, Deutschland; 4 Sachgebiet 10, Regierung der Oberpfalz, Regensburg, Deutschland

**Keywords:** SARS-CoV‑2, Massenanfall von Infizierten, Alten- und Pflegeeinrichtungen, Rettungsdienst, Krisenmanagement, Mass casualty incidents, Emergency medical services, Residential facilities, Disease outbreaks, SARS-CoV‑2

## Abstract

**Hintergrund:**

Während der Corona Virus Disease-2019(COVID-19)-Pandemie sind gehäuft Ausbrüche in stationären Pflege- und Betreuungseinrichtungen zu beobachten, die die ambulanten Strukturen überfordern und zu einer rettungsdienstlichen Großschadenslage aufwachsen. Standardisierte einsatztaktische Vorgaben wie für den Massenanfall von Verletzten (MANV) fehlen.

**Methoden:**

Anhand eines konkreten Fallberichts und der Literatur stellen die Autoren eine Managementstrategie für den Massenanfall von Erkrankten bzw. Infizierten (MANE/MANI) während der Severe Acute Respiratory Syndrome Corona Virus‑2(SARS-CoV‑2)-Pandemie vor und grenzen diese zur MANV-Einsatztaktik ab.

**Ergebnisse:**

Das Vorgehen bei MANI lässt sich in 3 Phasen einteilen und beginnt mit der akuten Gefahrenabwehr mit Sichtung, Stabilisierung kritischer Patienten und Transport der hospitalisierungspflichtigen Patienten. In Phase 2 werden die Betriebsbereitschaft der Einrichtung gesichert oder die Bewohner anderweitig untergebracht, falls das Personal in relevantem Ausmaß infiziert oder in Quarantäne ist. Die 3. Phase markiert die Rückkehr zum Regelbetrieb.

**Diskussion:**

Phase 1 orientiert sich an MANV-Grundsätzen, Phase 2 am Krankenhauskrisenmanagement. Die Vermeidung einer Evakuierung der Bewohner zur Entlastung der Krankenhäuser stellt dabei ein wichtiges Einsatzziel dar. Mangelnde Einsatz- und Übungspraxis mit derartigen Lagen, die begrenzte Anwendbarkeit von etablierten Vorsichtungsalgorithmen sowie der hohe Koordinationsbedarf stellen die Führungskräfte vor Herausforderungen.

**Schlussfolgerung:**

Das vorgestellte Phasenmodell stellt einen praktikablen, ganzheitlichen Ansatz zum erweiterten notfallmedizinischen Management von MANI-Einsatzlagen dar.

## Abstract (en)

**Background:**

During the coronavirus disease 2019 (COVID-19) pandemic, outbreaks in inpatient care facilities, which grow into a large-scale emergency scenario, are frequently observed. A standardized procedure analogous to algorithms for mass casualty incidents (MCI) is lacking.

**Methods:**

Based on a case report and the literature, the authors present a management strategy for infectious MCI during the severe acute respiratory syndrome coronavirus 2 (SARS-CoV-2) pandemic and distinguish it from traumatic MCI deployment tactics.

**Results:**

This management strategy can be divided into three phases, beginning with the acute emergency response including triage, stabilization of critical patients, and transport of patients requiring hospitalization. Phase 2 involves securing the facility’s operational readiness, or housing residents elsewhere in case staff are infected or quarantined to a relevant degree. Phase 3 marks the return to regular operations.

**Discussion:**

Phase 1 is based on usual MCI principles, phase 2 on hospital crisis management. Avoiding evacuation of residents to relieve hospitals is an important operational objective. The lack of mission and training experience with such situations, the limited applicability of established triage algorithms, and the need to coordinate a large number of participants pose challenges.

**Conclusion:**

This strategic model offers a practical, holistic approach to the management of infectious mass casualty scenarios in nursing facilities.

## Hintergrund und Fragestellung

Ausbrüche in Altenheimen und stationären Pflegeeinrichtungen stellen ein häufiges Phänomen während der Corona Virus Disease-2019(COVID-19)-Pandemie dar und sind mit hoher Morbidität und Mortalität assoziiert [20, 24]. Während bis dato ein Massenanfall von Erkrankten (MANE) bzw. Infizierten (MANI) ein seltenes Ereignis im Rettungsdienst darstellte [25], sehen sich die Sanitätseinsatzleitungen (San-EL) aktuell wiederholt mit MANI-Lagen in stationären Pflegeeinrichtungen konfrontiert. Durch die gleichzeitige Erkrankung bzw. Quarantänepflicht von sowohl Bewohnern als auch Personal reichen die Konsequenzen solcher Ereignisse bis hin zum völligen Zusammenbruch der Betriebsbereitschaft der jeweiligen Einrichtung. Gleichzeitig finden diese Ausbrüche im Kontext einer reduzierten Aufnahmebereitschaft der Krankenhäuser statt. Die einsatztaktischen Vorgehensweisen für den Massenanfall von Verletzten (MANV) lassen sich nur eingeschränkt auf diese Einsatzlagen übertragen.

## Studiendesign und Untersuchungsmethoden

Auf Basis der Erfahrungen aus 2 MANI-Einsätzen in Alten- bzw. Pflegeheimen stellen die Autoren ein Phasenmodell für das akute Management derartiger Ereignisse dar. Ergänzend wurde eine PubMed-Literaturrecherche mit den Medical Subject Headings „mass casualty incidents“, „emergency medical services“, „residential facilities“ und „disease outbreaks“ sowie eine selektive Literatursuche durchgeführt.

## Ergebnisse

### Fallbericht

In einer stationären Pflegeeinrichtung mit 47 Bewohnern wird nach vereinzelten COVID-19-Fällen eine Reihentestung durchgeführt. Während der Testphase liefert der Rettungsdienst bereits 5 symptomatische Patienten in Kliniken ein.

#### Tag 1 (Donnerstag)

In der Testung erweisen sich 37 der 42 verbliebenen Heimbewohner sowie 17 Mitarbeiter als „Severe Acute Respiratory Syndrome Corona Virus‑2“(SARS-CoV‑2)-positiv. Für weitere Beschäftigte wird als Kontaktpersonen der Kategorie I Quarantäne angeordnet. Dies führt dazu, dass nur eine einzige Pflegekraft zur Spätschicht erscheint. Über diese Situation wird die Katastrophenschutzbehörde (Führungsgruppe Katastrophenschutz [FüGK]) informiert, die um 14.30 Uhr die Entsendung der San-EL zur Lageerkundung veranlasst.

Der San-EL wird übergeben, dass mehrere Bewohner einen reduzierten Allgemeinzustand und Atembeschwerden aufwiesen. In einer ersten Lagemeldung werden daraufhin 2 Rettungstransportwägen (RTW), ein Notarzteinsatzfahrzeug (NEF) sowie die Unterstützungsgruppe San-EL angefordert.

In vollständiger Infektionsschutzausrüstung beginnen 3 Teams gegen 15.00 Uhr mit der Sichtung. Abweichend vom lokal etablierten Amberg-Schwandorf-Algorithmus für die Vorsichtung (ASAV; [12]) wird nach der COVID-19-Entscheidungshilfe der ÄLRD Bayern gesichtet [23, 27]. Das Sichtungsergebnis wird zusammen mit Vitalparametern und Temperatur auf Patientenanhängekarten dokumentiert.

Die Sichtung ist nach 90 min abgeschlossen. Nachalarmierte Rettungsmittel übernehmen den Transport von 8 hospitalisierungspflichtigen Patienten (einmal Sichtungskategorie [SK] I, 7‑mal SK II). Bei reduzierten Aufnahmekapazitäten müssen diese auf 3 Zielkliniken verteilt werden, was bis 17.30 Uhr abgeschlossen ist.

Parallel wird durch die FüGK festgestellt, dass der Träger für die nächsten 24 h nicht in der Lage ist, die Einrichtung personell zu besetzen. Aufgrund der großen Anzahl erkrankter Bewohner besteht zudem ein deutlich erhöhter Betreuungsbedarf. Mithilfe eines privaten ambulanten Pflegediensts kann schließlich der Personalbedarf bis zum nächsten Nachmittag gedeckt und eine Evakuierung des Heims abgewendet werden.

#### Tag 2 (Freitag)

Eine halbe Stunde vor der geplanten Übergabe teilt der Einrichtungsträger mit, dass die Übernahme des Betriebs nicht wie vereinbart erfolgen kann. Die FüGK entscheidet sich daraufhin erneut zur Alarmierung der San-EL. Vor Ort werden eine neuerliche Sichtung durchgeführt und wiederum 2 akute Kliniktransporte mittels RTW organisiert. Notdürftig kann, u. a. durch Unterstützung einer Hilfsorganisation und weiterer Freiwilliger, eine pflegerische Besetzung für die Nacht auf die Beine gestellt werden.

#### Tag 3 (Samstag)

Die FüGK entscheidet, den Betrieb im Rahmen einer Ersatzvornahme dem privaten Pflegedienst interimsweise zu übertragen. Vor der Übergabe an diesen am Abend erfolgt eine dritte Sichtungsrunde durch den Leitenden Notarzt (LNA) mit 2 weiteren resultierenden Klinikeinweisungen.

#### Tag 4 (Sonntag)

Der LNA führt über seine vor Ort befindliche Allgemeinarztpraxis die ärztliche Betreuung der Bewohner fort und verordnet insbesondere erforderliche Dauer- und Bedarfsmedikation, die durch eine lokale Apotheke ausgegeben wird. Abends kann die Einrichtung wieder in die reguläre ambulante Versorgung übergeben und der Einsatz endgültig abgeschlossen werden.

### Abgrenzung MANV vs. MANI

In der Literatur wird das Management von traumatisch bedingten Großschadenslagen umfassend behandelt [6, 18, 19], während sich zum Thema notfallmedizinische Einsatztaktik bei MANE/MANI keine spezifischen Einsatzalgorithmen finden. Gängige Sichtungskonzepte sind überwiegend für MANV-Lagen erstellt und evaluiert und können nicht ohne weiteres auf SARS-CoV-2-Infektionen übertragen werden. Die aktuelle Pandemiesituation stellt zudem erweiterte Anforderungen an die Einsatztaktik, die bei bisherigen Planungen nicht berücksichtigt wurden. Wesentliche Unterschiede zwischen MANV- und MANI-Lagen sind in Tab. 1 zusammengefasst.

**Table 1:** 

	MANV	MANI
Vorbereitung	Einsatzkonzepte etabliert, Übungs- und Einsatzerfahrung vorhanden	Keine etablierten Konzepte, kaum Übungs- und Einsatzerfahrung
Infrastruktur	Krankenhäuser i. d. R. voll leistungsfähig. Betreuungsbedarf für unverletzte Betroffene meist nur kurz	Krankenhäuser bereits mit COVID-19-Patienten belastet. In Heimen u. U. (aufwändigere) pflegerische Betreuung der verbleibenden Bewohner nicht gewährleistet
Dynamik	Meist singuläres Schadensereignis ohne wesentliche Dynamik, jedoch rasche Änderungen des Patientenzustands möglich. ⇒ Wiederholte Nachsichtungen in kurzen Zeitabständen ⇒ Ggf. Soforttransporte, sofern Patient nicht stabilisierbar	Schadenslage dynamisch durch neue oder progrediente klinische Symptomatik sowie Ausbreitung der Infektion. Patientenzustand weniger dynamisch. ⇒ Nachsichtungen in größeren Abständen über einen längeren Zeitraum ⇒ Soforttransporte seltener notwendig
Aufgaben	Notfallmedizinische Routineaufgaben, ggf. Massenanfallsversorgung mit kurzfristigem Abweichen von individualmedizinischen Standards	Wie bei MANV. Zusätzlich ggf. Übernahme pflegerischer Tätigkeiten
Sichtung	Etablierte, evaluierte Vorsichtungsalgorithmen. Zeitbedarf unter einer Minute pro Patient (von individueller SK abhängig; [16, 28]). SK IV nur im absoluten Ausnahmefall	Modifikation des Sichtungsvorgangs notwendig (Pulsoxymetrie und Körpertemperatur; neurologische Komponenten ggf. nicht aussagekräftig). Patientenwille kann zur SK IV führen. Höherer Zeitbedarf
Belastung der Einsatzkräfte	Psychische Belastung dominiert	Neben psychischem Stress (u. a. Angst vor eigener Ansteckung) zusätzliche Belastung durch Tragen der Schutzausrüstung

*MANV* Massenanfall von Verletzten, *MANI* Massenanfall von Infizierten, *COVID-19* Corona Virus Disease-2019, *SK* Sichtungskategorie

### Zielsetzung MANI-Management

Das MANI-Management in Rahmen der COVID-19-Pandemie geht über die akute Gefahrenabwehr hinaus und dient unter anderem dazu, ohnehin schon an der Grenze der Belastbarkeit agierende Krankenhäuser von vermeidbaren Patientenaufnahmen zu entlasten und eine Infektionsverschleppung in andere Einrichtungen zu verhindern. Wann immer möglich sollte die Evakuierung betroffener Einrichtungen trotz der Schwächung der Personaldecke durch Erkrankung oder Quarantänepflicht vermieden werden. Dies findet beim nachfolgenden Phasenmodell (Tab. 2) Berücksichtigung.

**Table 2:** 

	Zeithorizont	Maßnahmen	Zuständigkeiten
Phase 1: akute Gefahrenabwehr	Stunden	(Vor‑)Sichtung	San-EL/Rettungsdienst
		Kritische Patienten stabilisieren	
		Akuten Hospitalisierungsbedarf feststellen	
		Kliniktransporte organisieren	
Phase 2: Übergangsbetrieb sicherstellen	Stunden – Tage	Entscheidung über Evakuierung von Gesunden und/oder Infizierten	Heimleitung/Träger/San-EL/Kreisverwaltungsbehörde (FüGK, ÖGD, Heimaufsicht)/Fahrdienste
		Absonderungsmaßnahmen sicherstellen	
		Infektionsüberwachung gewährleisten	
		Pflegerische Übergangsbetreuung sicherstellen	
		Ambulante medizinische Versorgung sicherstellen (ärztliche Versorgung, Medikamente, Heilmittel)	
		Ggf. Transporte i. R. d. Evakuierung organisieren	
Phase 3: Regelbetrieb wiederherstellen	Tage – 1 Woche	Pflegerische Regelbetreuung wiederherstellen	Heimleitung/Träger/Kreisverwaltungsbehörde (FüGK, ÖGD, Heimaufsicht)/Fahrdienste
		Ambulante medizinische Versorgung sicherstellen	
		Absonderungsmaßnahmen konsequent fortführen	
		Infektionsüberwachung gewährleisten	
		Rücktransporte von Evakuierten organisieren	
		Reserven schaffen (Personal, Material)	

*San-EL* Sanitätseinsatzleitung, *FüGK* Führungsgruppe Katastrophenschutz, *ÖGD* öffentlicher Gesundheitsdienst

### Phase 1: akute Gefahrenabwehr

Die Phase 1 orientiert sich weitgehend an den bei MANV-Lagen üblichen Einsatzgrundsätzen. Nach einem ersten Lageüberblick und Kräfteabgleich werden anhand einer (Vor‑)Sichtung der sofortige Behandlungs- und Transportbedarf festgestellt und entsprechende rettungsdienstliche Ressourcen zugeteilt. Im Anschluss werden weniger kritische, aber dennoch hospitalisierungspflichtige Patienten identifiziert und deren Klinikaufnahme organisiert. Besonderes Augenmerk wird auf die Sicherheit der Einsatzkräfte gelegt, die insbesondere durch fachgerechte Durchführung von Barrieremaßnahmen (persönliche Schutzausrüstung, Desinfektion) gewährleistet wird. Bei längerdauernden Einsätzen muss die Phase 1 unter Umständen mehrfach durchlaufen werden (s. Fallbeispiel).

#### (Vor‑)Sichtung und Versorgung kritischer Patienten

Im Rahmen einer nichtärztlichen Vorsichtung mittels starrer Algorithmen oder einer ersten ärztlichen Sichtung werden Patienten mit akuter Störung der Vitalfunktionen und sofortigem Behandlungsbedarf (SK I, „rot“, Sofortbehandlung) identifiziert [9]. Bei der Vorsichtung wird man aus pragmatischen Erwägungen häufig nicht umhinkommen, den jeweiligen für den MANV-Fall etablierten Vorsichtungsalgorithmus anzuwenden, auch wenn dieser regelhaft nur bedingt für internistische/infektiologische Krankheitsbilder geeignet ist. Umso wichtiger ist die nachfolgende ärztliche Sichtung, die spätestens im Rahmen der Entscheidungsfindung über den Hospitalisierungsbedarf (s. unten) erfolgt. Neben den rein medizinischen Kriterien sollte auch das Vorhandensein eventueller Patientenverfügungen berücksichtigt werden, sodass bei schwerstem Verlauf und gleichzeitiger Ablehnung einer intensivmedizinischen Therapie auch die Einordnung in SK IV (palliative Behandlung) zu erwägen ist.

Im obigen Fallbeispiel wurde die erste Sichtung nicht nach dem Vorsichtungsalgorithmus, sondern nach der Entscheidungshilfe Hospitalisierung der Ärztlichen Leiter Rettungsdienst (ÄLRD) Bayern [23, 27] vorgenommen und um die Messung von Vitalparametern und Temperatur ergänzt. Dies führte, zusammen mit den notwendigen Hygienemaßnahmen, zu einem unerwartet hohen Zeitbedarf (über 6 min pro Patient).

In SK I gesichtete Patienten werden schnellstmöglich notfallmedizinisch stabilisiert und, sobald entsprechende Transport- und Aufnahmekapazitäten bereitgestellt wurden, in geeignete Krankenhäuser verbracht. Die Klinikzuweisung erfolgt analog zu den üblichen rettungsdienstlichen Vorgehensweisen mit entsprechender Voranmeldung (z. B. über die Leitstelle oder einen elektronischen Betten- und Behandlungskapazitätennachweis [14]). Soforttransporte, wie sie nach schweren Traumata mitunter notwendig sind, dürften die Ausnahme darstellen.

#### Hospitalisierungsbedarf feststellen und realisieren

Der Hospitalisierungsbedarf wird nach medizinischen Kriterien festgestellt und ist eine ärztliche Entscheidung, die auf Grundlage von entsprechenden Entscheidungshilfen getroffen werden kann [13, 23, 27]. Bei der Beurteilung kann die Einbeziehung von allgemeinmedizinischem Sachverstand bzw. die Abstimmung mit den weiterbetreuenden ambulanten ärztlichen und pflegerischen Versorgungsstrukturen von Vorteil sein.

Da es sich bei diesem Patientenkollektiv nicht mehr um kritische Patienten handelt, kann die Bettenzuweisung unter Einbeziehung der zuständigen Führungsebenen für die übergeordnete Bettenkapazitätssteuerung erfolgen [11]. Die Durchführung der Transporte erfolgt durch den Rettungsdienst, ggf. unter Nutzung von Sonderfahrzeugen und Kohortentransporten, um die Einsatzmittel der Regelvorhaltung zu entlasten.

### Phase 2: Übergangsbetrieb sicherstellen

Während bei präklinischen MANV-Lagen der Einsatz mit Abschluss der ersten Phase und Räumung der Einsatzstelle in der Regel abgeschlossen ist, stellt sich bei Ausbruchsgeschehen, wie im obigen Fallbeispiel dargestellt, ein nachfolgendes Problem: Wenn das Pflegepersonal der Einrichtung zu einem erheblichen Anteil selbst ausfällt, kann unter Umständen die Versorgung der verbliebenen Bewohner der Einrichtung nicht mehr gewährleistet werden. Erschwerend kommt hinzu, dass auch nichthospitalisierte COVID-19-Erkrankte einen deutlich höheren Pflegeaufwand benötigen, sodass die reguläre Personalbesetzung gegebenenfalls nicht ausreicht. Dies führt im ungünstigsten Fall zu einer Evakuierung der gesamten Einrichtung verbunden mit der Verlegung einer größeren Anzahl COVID-19-(Verdachts‑)Fällen in andere Einrichtungen (Pflegeeinrichtungen oder Krankenhäuser). In Zeiten ohnehin angespannter Belegungssituationen sollte dies tunlichst vermieden werden, was nur im Zusammenspiel von Träger bzw. Heimleitung, Kreisverwaltungsbehörden (Katastrophenstab, öffentlicher Gesundheitsdienst [ÖGD], Heimaufsicht) und im Ausnahmefall der San-EL und des Katastrophenschutzes bewerkstelligt wird. Insbesondere in Bezug auf administrativ-organisatorische Weichenstellungen (Personaluntergrenzen etc.) ist eine enge Abstimmung mit der Aufsichtsbehörde, in diesem Fall der Heimaufsicht, anzustreben.

#### Entscheidung über Evakuierung

**Figure 1:**
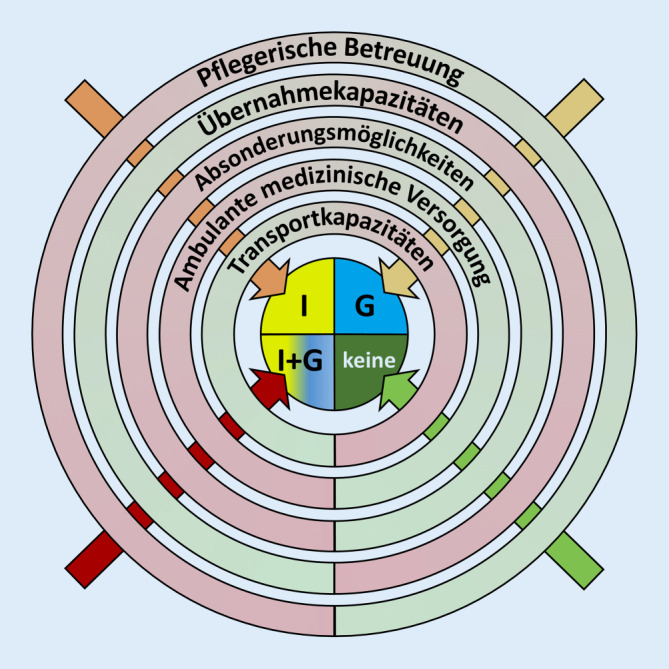

Für die Entscheidung, in welchem Umfang ein Weiterbetrieb der Einrichtung möglich gemacht werden kann bzw. ob die (Teil‑)Evakuierung von Gesunden und/oder Erkrankten bzw. Infizierten erfolgen muss, können die Kriterien in Tab. 3 sowie Abb. 1 herangezogen werden.

**Table 3:** 

*Pflegerische Betreuung sichergestellt?*
*Krankenhauskapazität vorhanden? Sonstige Übernahmemöglichkeiten vorhanden?*
Absonderungsmöglichkeiten vorhanden oder zeitgerecht organisierbar (Räumlichkeiten, PSA, Hygieneartikel)?
Ambulante medizinische Betreuung gewährleistet?
Transportkapazität vorhanden?

#### Pflegerische Übergangsbetreuung sicherstellen

Sofern die pflegerische Betreuung nicht durch das verbliebene heimeigene Personal geleistet werden kann, muss dies anderweitig organisiert werden. Größere Träger können ggf. Pflegende aus anderen eigenen Einrichtungen zusammenziehen. Ansonsten ist zu prüfen, ob zur Unterstützung Freiwillige (z. B. aus dem Pflegepool der Vereinigung der Pflegenden in Bayern), Personal anderer Träger, Ehrenamtliche der Hilfsorganisationen oder Bundeswehrangehörige (nur im Katastrophenfall) rekrutiert werden können. Auch ist der Einsatz von asymptomatischem infiziertem Pflegepersonal nach den Maßgaben des Robert Koch-Instituts zu erwägen. Im Extremfall kann der Betrieb übergangsweise an einen anderen, leistungsfähigen Betreiber übergeben werden. Ein ausreichender Fachkräfteanteil muss jeweils sicherstellt sein, der jedoch kurzfristig die vorgeschriebene Fachkraftquote unterschreiten kann.

#### Absonderungsmaßnahmen und Infektionsüberwachung sicherstellen

Für die verbleibenden Bewohner sind in Absprache mit dem ÖGD die notwendigen Absonderungsmaßnahmen (Isolation, Quarantäne) sicherzustellen. Hierfür müssen gewisse personelle und bauliche Anforderungen erfüllt sowie ausreichend Hygienematerial (persönliche Schutzausrüstung wie Schutzmasken, -brillen und -anzüge, Desinfektionsmittel) verfügbar sein. Es ist häufig sinnvoll, positiv getestete Bewohner in einen abgetrennten Bereich zusammenzulegen, um diese räumlich von den Gesunden zu isolieren. Durch eine engmaschige strukturierte klinische Überwachung der Bewohner kann auf neu symptomatische oder sich klinisch verschlechternde Patienten rechtzeitig reagiert werden.

#### Ambulante medizinische Versorgung sicherstellen

Neben der pflegerischen ist auch die ambulante hausärztliche Betreuung von großer Bedeutung für die Betriebsbereitschaft der Einrichtung. Diese kann u. a. durch Arbeitsüberlastung oder eigene Krankheits- bzw. Quarantänefälle der ambulanten Arztpraxen gefährdet sein. Ebenso ist auch die Versorgung mit Arznei- und Heilmitteln (insbesondere Sauerstoff) zu gewährleisten.

In Bayern wurde die Sicherstellung der ambulanten ärztlichen Versorgung im Rahmen des Krisenfalls auf der Ebene der Kreisverwaltungsbehörden in die Hände von eigens bestellten Versorgungsärzten [3] bzw. koordinierenden Ärzten [5] gelegt. Bewährt hat sich die interimsweise Beauftragung von Heimärzten, die sich um die Betreuung sämtlicher Bewohner kümmern. Die freie Arztwahl ist jedoch zu gewährleisten; insbesondere können keine Besuche anderer Ärzte ausgeschlossen werden.

#### Transporte im Rahmen der Evakuierung organisieren

Sofern die Entscheidung zugunsten einer (Teil‑)Evakuierung gefallen ist, müssen die entsprechenden Transporte organisiert werden. Bewohner, die aufgrund ihres klinischen Zustands nicht auf einen qualifizierten Krankentransport angewiesen sind, sollten möglichst mit anderweitigen Fahrdiensten befördert werden, um den Rettungsdienst zu entlasten. Ist dies nicht möglich, sind Kohortentransporte zu erwägen.

### Phase 3: Regelbetrieb wiederherstellen

Träger und Leitung der Einrichtung sind gefordert, in Abstimmung mit dem ÖGD und der Heimaufsicht so rasch wie möglich die Voraussetzungen für die Wiederaufnahme des Regelbetriebs zu schaffen. Neben einem funktionierenden Dienstplan mit eigenem Pflegepersonal sind die ambulante medizinische Versorgung sowie angeordnete Absonderungs- und Infektionsüberwachungsmaßnahmen (Symptomverfolgung, Nachtestungen) sicherzustellen.

Im Anschluss kann sukzessive mit dem Rücktransport der ausgelagerten bzw. entlassfähigen Bewohner begonnen werden. Bezüglich der Transporte sollten o. g. Prinzipien beachtet werden.

Auch den eingesetzten Kräften des Rettungsdiensts sollte trotz stringenter Barrieremaßnahmen eine Selbstüberwachung auf Symptome und eine Virustestung angeraten und ermöglicht werden.

## Diskussion

Die SARS-CoV-2-Pandemie stellt einen globalen Gesundheitsnotstand dar und sprengt gängige Schweregradeinteilungen von Großschadenslagen und Katastrophen [10]. COVID-19-Ausbrüche in stationären Pflegeeinrichtungen sind ein häufiges Phänomen während der Pandemie. Die hier dargestellte Einsatzstrategie stellt einen praktikablen, ganzheitlichen Ansatz zum erweiterten notfallmedizinischen Management solcher Lagen dar.

Die erste Phase lehnt sich dabei an die bekannten Einsatzgrundsätze der MANV-Versorgung an, sodass die Anwendung für die rettungsdienstlichen Einsatz- und Führungskräfte kein wesentliches Abweichen von etablierten Standards bedeutet. Dagegen orientiert sich Phase 2 mehr an Strategien des pandemiebezogenen Krankenhauskrisenmanagements mit den Stellgrößen Personal, Ressourcen und Raum [29–31]. Hier steht das Ziel im Vordergrund, in der betroffenen Pflegeeinrichtung mindestens das Betreuungsniveau der kompensierten Krisenversorgung zu erreichen, um den Betrieb aufrechterhalten zu können.

Ein vergleichbares Phasenmodell wurde bei einem Ausbruch in einem kanadischen Pflegeheim im Rahmen eines COVID-19-Ausbruchs angewendet. Hier erfolgte die organisatorische Unterstützung jedoch nicht durch den Rettungsdienst, sondern durch ein Krankenhaus [26].

Anders als bei den häufigeren MANV-Lagen kann der Rettungsdienst zu diesen speziellen MANI-Situationen wenig Übungs- und Praxiserfahrung vorweisen und es sind keine spezifischen einsatztaktischen Handlungsempfehlungen verfügbar. Ferner kann der Einsatz nicht in jedem Fall nach der ersten Einsatzphase als abgeschlossen betrachtet werden. Im Gegensatz zu MANI-Lagen außerhalb der Pandemie stellt das Erkennen der infektiösen Genese hingegen weniger Schwierigkeiten dar und die Einsatzkräfte sind in Bezug auf die Vorhaltung der erforderlichen Schutzausrüstung und den richtigen Umgang damit gerüstet. Anders als bei auf unklare biologische oder auf sonstige gefährliche Agenzien zurückzuführenden Gefahrenlagen sind zudem keine über die üblichen Hygienevorkehrungen hinausgehenden Dekontaminationsmaßnahmen erforderlich.

Während für traumatologische Großschadenslagen eine Reihe von konkreten Handlungsempfehlungen vorliegt [6, 18, 19], sucht man diese für den MANE‑/MANI-Fall vergeblich.

Die in der Bundesrepublik gängigen Vorsichtungsalgorithmen wurden überwiegend für chirurgische Patienten entwickelt und evaluiert [2, 7, 12, 16–18, 22, 28] und zeigen bei internistischen Patienten eine geringere Treffsicherheit [1]. Lediglich das Primäre Ranking zur initialen Orientierung im Rettungsdienst (PRIOR; [8]) ist explizit auch für internistische Patienten vorgesehen und kann bei diesem Kollektiv rote Patienten sensitiver identifizieren, neigt jedoch zu relevanter Übertriage [1]. Während in der Traumasichtung die motorische Komponente des GCS die größte Vorhersagekraft für schwerverletzte Patienten aufweist [15], ist dieser Parameter bei vorbestehend kognitiv oder motorisch beeinträchtigen Patienten wenig aussagekräftig, was die Anwendbarkeit von üblichen Vorsichtungsalgorithmen einschränkt. Die Bedeutung der ärztlichen Sichtung sei nochmals hervorgehoben.

Bei fehlendem Behandlungswillen kann die Eingruppierung in die Sichtungskategorie IV mit nachfolgender palliativer Behandlung gerechtfertigt sein. Sofern eine entsprechend dokumentierte und kommunizierte Festlegung des Patientenwillens im Sinne eines *Advance Care Planing* besteht, ist dies für den Akutfall von Vorteil [21].

Das vorgestellte Fallbeispiel stellt sicherlich einen Extremfall mit komplettem Zusammenbruch der Betriebsbereitschaft der Pflegeeinrichtung dar. Eine Evakuierung konnte nur durch einen langdauernden Einsatz der San-EL und ein intensives Zusammenwirken aller Beteiligten letztlich mithilfe eines externen Pflegeunternehmens verhindert werden. In den meisten Fällen wird sich die betroffene Institution jedoch unmittelbar von der ersten Akutphase wieder in den Regelbetrieb überführen lassen, ohne dass es einer Übergangsphase bedarf. Umgekehrt ist ebenso denkbar, dass es keines Einsatzes der San-EL bedarf, sondern die Phasen 2 und 3 durch Heimleitung, niedergelassene Ärzteschaft und ggf. ÖGD bewältigt werden. Die beschriebene Einsatztaktik könnte auch auf Ausbrüche in anderen Einrichtungen, wie in Krankenhäusern, Reha-Kliniken oder Justizvollzugsanstalten, angewendet werden.

In komplexen Lagen wie dieser sind eine standardisierte Führungsstruktur und ein eingespielter Führungsablauf unverzichtbare Voraussetzungen für das Gelingen des Einsatzes [10]. Einen erheblichen Vorteil stellte im Einsatzbeispiel der zum Einsatzzeitpunkt bereits ausgerufene Katastrophenfall dar, da die behördlichen Führungsstrukturen (FüGK) schon etabliert waren und steuernd eingreifen konnten. In einer Ad-hoc-Lage ohne Katastrophenfall ist die Einbeziehung einer örtlichen Einsatzleitung (ÖEL) nach Art. 15 Bayerisches Katastrophenschutzgesetz erwägenswert, die durch ihre weitreichenden Befugnisse eine Gesamtkoordination der einzelnen Akteure leisten kann.

## Schlussfolgerung

Das vorgestellte Phasenmodell stellt das akute Management von COVID-19-Ausbrüchen in Alten- und Pflegeheimen dar und zeigt die Unterschiede zu MANV-Lagen auf. Insbesondere im Bereich der (Vor‑)Sichtung muss ggf. von etablierten Konzepten abgewichen werden. Nach der Akutphase steht bei kritischer Einschränkung der Personaldecke der Weiterbetrieb der Einrichtung im Fokus der Bemühungen. In Bayern werden die Katastrophenstäbe in Zukunft durch einen Pflegeleiter FüGK verstärkt, der übergeordnete Koordinationsaufgaben im Krisenmanagement der Pflegeeinrichtungen übernehmen soll [4]. Außerhalb des Katastrophenfalls sollte eine ÖEL einbezogen werden, die im Idealfall seitens des örtlichen Einsatzleiters mit einer medizinisch versierten Person (LNA, Organisatorischer Leiter Rettungsdienst) besetzt wird. Die aus der weltumspannenden Infektionslage gewonnen Erkenntnisse stellen eine wichtige Basis für die zukünftige Ausrichtung von sowohl Katastrophenverhütung („mitigation“) als auch Vorbereitung („preparedness“) dar, um für die nächste globale Pandemie besser gerüstet zu sein [10].

## Fazit für die Praxis

- Rettungsdienst und Katastrophenschutz sehen sich während der Corona Virus Disease-2019(COVID-19)-Pandemie mit Massenanfallslagen von „Severe Acute Respiratory Syndrome Corona Virus‑2“(SARS-CoV-2)-Infizierten in Alten- und Pflegeheimen konfrontiert.
- Hierbei muss von der üblichen Einsatztaktik für Massenanfallslagen abgewichen werden.
- Die Vermeidung einer Evakuierung zur Entlastung der Krankenhäuser stellt ein wichtiges Einsatzziel dar.
